# PD-L1-positive circulating tumor cells associate with tumor malignancy and impaired circulating immunity in patients with gastrointestinal tumors

**DOI:** 10.1038/s41598-026-43324-y

**Published:** 2026-05-07

**Authors:** Leon Jonathan Kusterer, Matthias Reeh, Yogesh Kumar, Till Clauditz, Anna Woestemeier, Klaus Pantel, Nathaniel Melling, Daniel Perez, Jakob Izbicki, Sabine Riethdorf, Leonie Konczalla

**Affiliations:** 1 Department of General, Visceral and Thoracic Surgery, University Medical Centre Hamburg-Eppendorf, 20246 Hamburg, Germany; 2 Mildred Scheel Cancer Career Center HaTriCS4, University Medical Center Hamburg-Eppendorf, Hamburg, Germany; 3 Department of General, Visceral and Thoracic Surgery, Marien Hospital Hamburg, 22087 Hamburg, Germany; 4 Department of General and Visceral Surgery, Asklepios Clinic Altona, 22763 Hamburg, Germany; 5 Institute of Pathology, Molecular Pathology and Cytopathology, University Medical Centre Hamburg-Eppendorf, 20246 Hamburg, Germany; 6 Institute of Tumor Biology, University Medical Centre Hamburg-Eppendorf, 20246 Hamburg, Germany; 7 Centre of Biomedical Research, Department of Data Sciences, 226001 Lucknow, India

**Keywords:** Biomarkers, Cancer, Immunology, Oncology

## Abstract

Immunotherapies such as anti–PD-L1 antibodies have transformed cancer treatment, but their effectiveness in gastrointestinal tumors remains limited. One reason may be the absence of reliable prognostic markers that can identify patients at risk and those likely to benefit from such therapies. Circulating tumor cells (CTCs) have therefore gained attention as potential clinical markers for assessing tumor burden and immune status. In this study, 105 patients with esophageal, gastric, or colorectal cancer were enrolled preoperatively. CTCs were isolated and enumerated from venous blood using CellSearch, and PD-L1 expression was assessed. Lymphocytes were enriched via Percoll gradient centrifugation and analyzed by multiparametric flow cytometry. Statistical associations were evaluated using Chi-square tests. CTCs were detected in 16.2% of patients, and among these, 35.3% exhibited PD-L1^+^CTCs. The presence of PD-L1^+^CTCs correlated with indicators of advanced disease, including tumor stage, metastasis, and vascular invasion. Additionally, patients who were CTC-positive, and especially those with PD-L1^+^CTCs, showed reduced immunocompetence, reflected by lower immune cell frequencies, suggesting a weakened systemic immune response. These findings indicate that PD-L1^+^CTCs reflect tumor-driven immune suppression and may facilitate metastatic spread. Therefore, PD-L1^+^CTCs should be considered as promising prognostic biomarkers and may provide a rationale for early immunotherapy.

**Supplementary Information:**

The online version contains supplementary material available at 10.1038/s41598-026-43324-y.

## Introduction

Gastrointestinal (GI) carcinomas represent the most common group of malignancies, with 3.4 million cancer-related deaths annually worldwide^1^. This indicates the great need for improved therapeutic as well as prognostic strategies and techniques. Regarding prognostic markers and treatment monitoring, the use of liquid biopsies can be a promising and innovative field in oncological treatment^2^.

In liquid biopsies, circulating tumor cells (CTCs) can be detected in the bloodstream of patients, which are single cancer cells that disseminate from the primary tumor site or metastases, pass through the vascular system, and migrate to distant organs. Those CTCs are considered an indicator of residual disease after therapy and are associated with an increased risk of metastasis and tumor recurrence^3^. The prognostic impact of CTCs has repeatedly been demonstrated, also by the development of sensitive detection protocols, such as CellSearch, in many types of cancer^4,31^.

As soon as the cancer cells leave the tumor microenvironment, the CTCs are exposed to the immune surveillance of the body. Although CTCs are exposed to potential immune-mediated cell death, these cells manage to evade the immune system of the host. Yet, little is known about the exact escape mechanisms. The immune system, in general, has a fundamental role in the detection and elimination of cancer cells in the primary tumor site and the blood, respectively. It is known that in tumor tissue, cancer cells can create a specific microenvironment by inhibiting the effector functions of the immune system, leading to T-cell exhaustion and senescence^5^. One important trigger to create T-cell exhaustion is the ligation of co-inhibitory receptors (CIRs), such as the programmed cell death protein-1 (PD-1) and the respective ligand, PD-L1^6^. The PD-1-PD-L1 interaction has been the focus of intensive research to develop new immunotherapeutic approaches. Recently, immunotherapies targeting CIRs have revolutionized the treatment of many types of cancers by unleashing the power of cytotoxic CD8^+^ T-cells and thereby empowering the endogenous immune system to kill tumor cells and cure cancer patients. However, in most gastrointestinal tumors, like colorectal cancer (CRC) and esophageal cancer (EC), immunotherapies such as Pembrolizumab (anti-PD-1) did not meet expectations with only a minor cohort of patients benefiting from the therapy^7^. Nonetheless immunotherapy has become a standard component of treatment for various gastrointestinal cancers. Particularly, checkpoint inhibitors such as anti-PD-1 and anti-PD-L1 therapies have shown efficacy in microsatellite instability-high (MSI-high) colorectal cancers and in gastroesophageal cancers with high PD-L1 expression, measured by the Combined Positive Score (CPS). These biomarkers, MSI and CPS, are currently the most established predictors of response to immunotherapy and are routinely used in clinical practice ^32–34^. This underscores the necessity for validated, reliable biomarkers, especially in heterogeneous tumors such as gastrointestinal malignancies.

However, so far these analyses have focused on the interactions in the tumor tissue, but not on interactions that already appear in the bloodstream, for example with CTCs. Whether PD-1 ligation also contributes to the CTC escape in the bloodstream, is not fully understood yet. However, the following suppression of anti-tumorigenic T-cells could facilitate the immune evasion of CTCs^8^. To this end, an immunotherapeutic strategy to prevent CTC-driven immunosuppression could open a new and effective field of therapy.

Recent studies have shown that CTCs of Non-Small Cell Lung Cancer (NSCLC) or breast and bladder cancer can express PD-L1 ^9–11^. However, in gastrointestinal cancer patients, PD-L1 expression on CTCs has never been tested before.

In the following, we show that also gastrointestinal cancer patients show PD-L1 expression on CTCs which correlates with advanced tumor progress. We hypothesize that PD-L1^+^ CTCs are more likely to seed distant metastases and cancer recurrence by contributing to attenuating the immune response in the bloodstream. Therefore, we propose PD-L1^+^ CTCs as a prognostic marker and as a rationale for immunotherapeutic regimes in those patients.

## Results

### CTC detection and their expression of PD-L1 predicts advanced tumor stage in gastrointestinal cancer patients

**Fig. 1:**
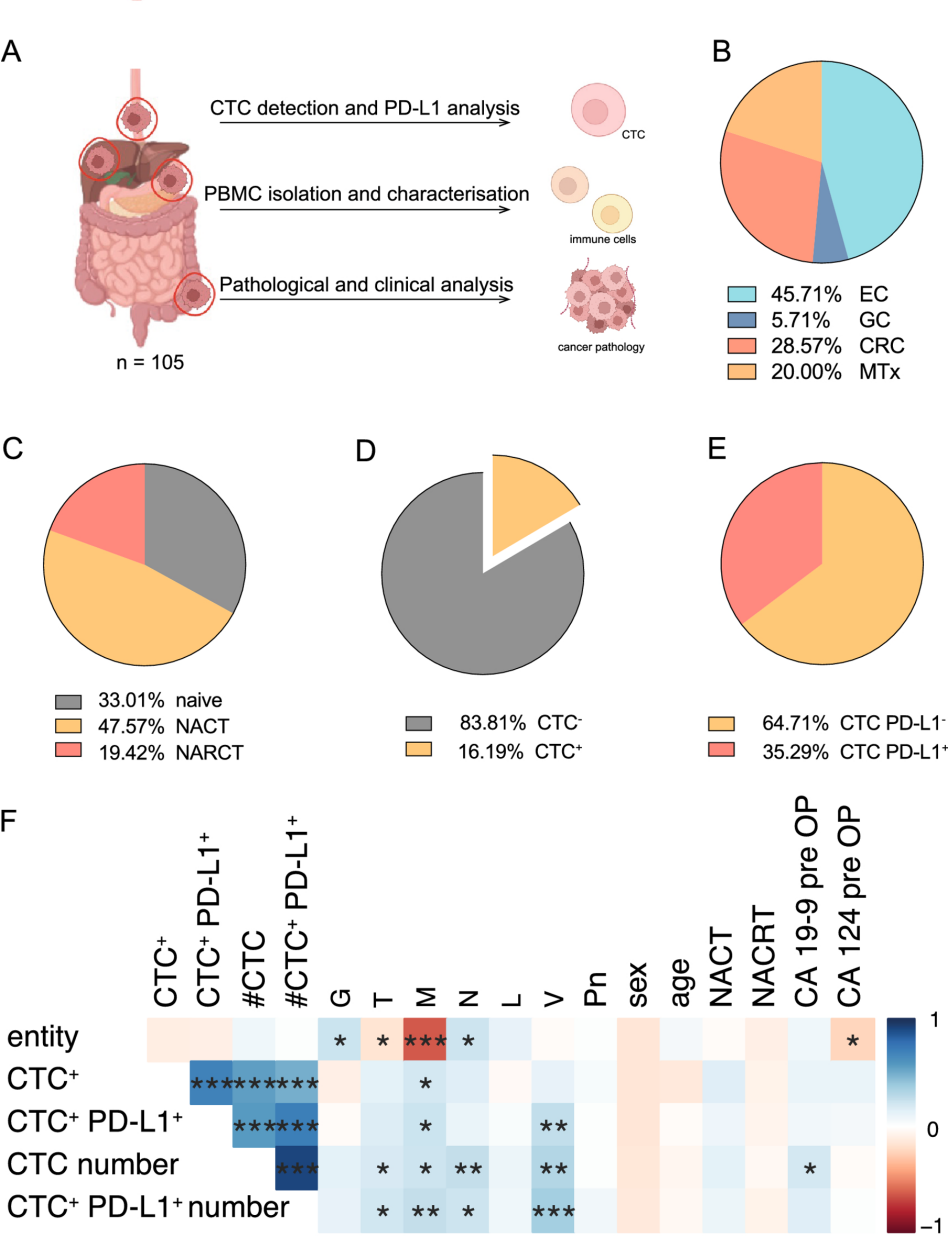
(**A**) Experimental setup and sample processing of 105 cancer patients; (**B**) Description of the patient population divided by cancer entity (45.71% Esophageal adenocarcinoma (EC), 5.71% Gastric cancer (GC), 28.57% Colorectal cancer (CRC), 20.00% Liver metastasis of CRC (MTx) (**C**) primary (33.01% treatment naive) and neoadjuvant radio-chemotherapy treated patients (47.57% Neoadjuvant chemotherapy treated (NACT); 19.42% Neoadjuvant radio chemotherapy treated (NARCT)) (**D**) as well as the proportion of CTC-positive patients (83.18% CTC^-^, 16.19% CTC^+^) of whom (**E**) 35.92% were PD-L1-positive CTCs (64.71% PD-L1^-^ CTCs, 35.29% PD-L1 + CTCs). (**F**) Statistical correlation (χ^2^ test) between patient characteristics, histopathological tumor classification, and CTC burden; showing a positive correlation between CTC detection as well as PD-L1 expression with the tumor stage, irrespectively of tumor marker, sex, age or neoadjuvant (radio) chemotherapy.

Peripheral blood samples were collected from 105 patients with gastrointestinal cancer on the day of and prior to surgery. Subsequently, blood samples were screened for CTCs, and the composition of white blood cells, especially the T lymphocytes, was determined. Those were characterized and the tumor samples were evaluated for immune infiltration and expression of the PD-1 (Fig. 1a). 

Patients’ collective resumed of 48 (45.71%) patients with esophageal adenocarcinoma (EC), 6 (5.71%) patients with gastric cancer (GC), 30 (28.57%) patients with colorectal cancer (CRC), and 21 (20%) patients with CRC liver metastases (Fig. 1b). One-third, 34.28% of the patients were chemotherapy naïve, while 46.67% received neoadjuvant chemotherapy treatment and an additional 19.05% received neoadjuvant radio-chemotherapy (Fig. 1c). In this patient cohort, cancer incidences varied across the different entities. The number of included patients reflects the distribution of these diagnoses and thus represents the cases requiring treatment.

CTCs were detected in 17 (16.19%) of 105 patients with an average of 3 CTCs per 7 ml blood, ranging from 1 to 17 (Fig. 1d). Out of these patients, 6 had CTCs (35.29%) which expressed PD-L1 (Table 1).

**Table 1:** Summary of detected CTCs and CTCs with PD-L1 expression (CTC^+^PD-L1^+^) in total as well as the average number and standard error of CTCs, and CTC^+^PD-L1^+^ per patient.

	CTC^NEG^	CTC^+^	CTC^+^PD-L1^+^
Number of patients	88	17	6
Number of CTCs		46	22
Average / patient		2.76 (± 1.79)	3.66(± 0.96)

Remarkably, neither the detection of CTCs in general nor their expression of PD-L1 correlated with the cancer entity, thus for the first statistical analysis, we considered the different tumor types as an aggregate of gastrointestinal tumors (Fig. 1f).

This analysis revealed a strong correlation between the CTC status of the cancer patients, concerning the general presence of CTCs, as well as their expression of PD-L1, with the overall tumor burden of the patients, especially Tumor stage (T)(pT_PD-L1+_ = 0.09; pT_#PD-L1+_ = 0.02), metastases in distant organs (M)(pM_PD-L1+_ = 0.01; pM_#PD-L1+_ = 0.007) or lymph nodes (N)(pN_PD-L1+_ = 0.27; pN_#PD-L1+_ = 0.01), as well as PD-L1^+^ CTCs with the microscopical tumor invasion of the blood vessels (V)(pV_PD-L1+_ = 0.006; pV_#PD-L1+_ = 0.0001). In contrast, general clinical characteristics such as sex, age, neoadjuvant therapy, or even the status of pre-operative tumor markers revealed no correlation with the patients’ CTC status (Fig. 1f and Table 2; Supp Fig. 1 a-d).

**Table 2:** Patient characteristics with averages and standard error of TNM classification, as well as patient numbers with mutations, positive tumor marker values, and neoadjuvant therapy. No statistical differences among patient groups were found (t-test).

	All	EC	GC	CRC	MTx
Entity	105	48	6	30	21
G (Grading)	2.15 (± 0.04)	2.27 (± 0.08)	2.25 (± 0.13)	2.04 (± 0.04)	2.04 (± 0.07)
T (Tumor)	2.58 (± 0.1)	2.1 (± 0.16)	3 (± 0.17)	2.89 (± 0.16)	2.83 (± 0.2)
M (Metastases)	0.31 (± 0.04)	0 (± 0)	0.25 (± 0.13)	0.25 (± 0.08)	1 (± 0)
N (Nodes)	0.89 (± 0.1)	1.145 (± 0.16)	1.33 (± 0.36)	0.36 (± 0.12)	0.83 (± 0.18)
L (lymphatic vessels)	0.39 (± 0.05)	0.41 (± 0.08)	0.58 (± 0.15)	0.29 (± 0.09)	0.38 (± 0.1)
V (Vascular)	0.14 (± 0.03)	0.12 (± 0.05)	0.25 (± 0.13)	0.04 (± 0.09)	0.25 (± 0.09)
Pn (Nerves)	0.2 (± 0.04)	0.2 (± 0.06)	0.33 (± 0.14)	0.071 (± 0.05)	0.30 (± 0.09)
sex	37 F 68 M	10 F 38 M	3 F 3 M	15 F 15 M	9 F 12 M
Age [years]	61.93 (± 1.13)	66.02 ± 1.4)	56.83 (± 2.17)	59.46 (± 2.64)	60.71 (± 2.65)
KRAS mutation	25	–	–	16	9
BRAF mutation	3	–	–	3	0
Her2 mutation	7	3	2	1	1
MSI high	8	0	2	4	2
CA-19-9 pre OP (> 37 kU/l)	15	7	2	4	2
CA-19-9 post OP (> 37 kU/l))	5	2	1	1	1
CEA pre OP (> 6,5 µg/L)	49	19	3	10	17
CEA post OP (> 6,5 µg/L)	12	4	0	1	7
neoadjuvant chemotherapy	70	28	9	11	22
neoadjuvant radiation	20	9	–	6	5

### PD-L1 expression on CTCs favors growth of distant metastases

**Fig. 2:**
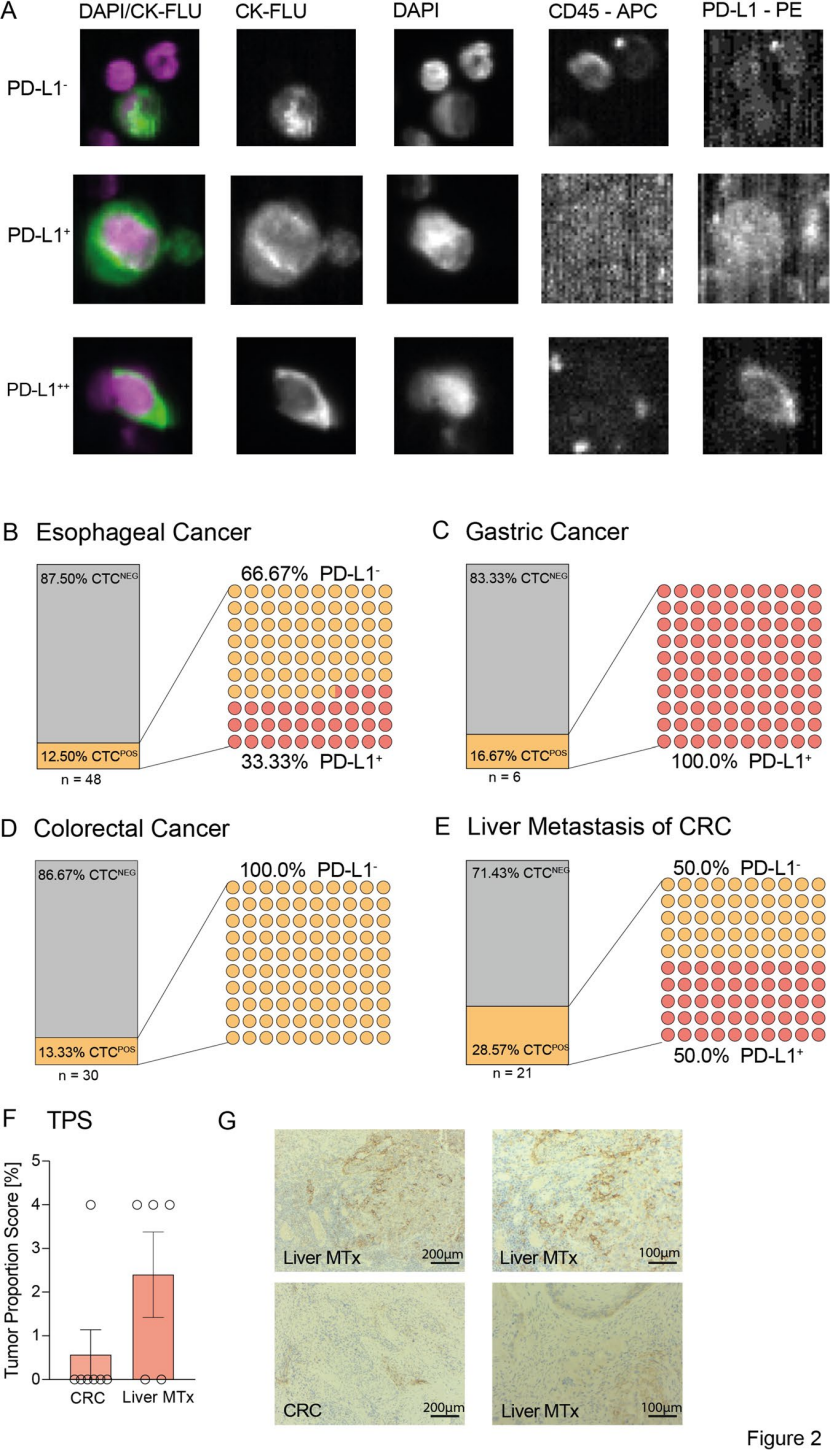
(**A**) CTCs without (upper cell), with weak (middle cell) and with moderate (bottom cell) PD-L1 expression. DAPI: 4′,6-diamidino-2-phenylindole; CK: cytokeratin; FLU: fluorescein; APC: allophycocyanin; PE: phycoerythrin. (**B–E**) The proportion of CTC-positive and CTC-negative patients, as well as their PD-L1 status, divided by diagnosis ((**B**) esophageal cancer (**C**) gastric cancer (**D**) colorectal cancer (**E**) liver metastasis). (**F**) Tumor proportion score (TPS) in CRC or liver metastasis tissue of patients with PD-L1^+^CTCs, shows a higher expression of PD-L1 in the tissue of metastases. (**G**) Histopathological staining: upper row: Liver metastasis of caecum carcinoma left 10x/ right 20 × magnification with positive PD-L1 staining with partial staining of tumor cells (TPS: 1–4) and immune cells (IC: 5–9) (CPS: 13) lower row: left Rectum carcinoma 20 × magnification with positive PD-L1 staining of tumor-associated lymphocytes (IC: 15) with negativity of carcinoma cells (TPS < 1) (CPS: 15); right: Metastasis of rectum carcinoma 20 × magnification with positive PD-L1 staining in 1–4% of tumor and immune cells (TPS + IC: 1–4, CPS: 7).

We analyzed the CTCs for the expression of PD-L1 by immunofluorescence (Fig. 2a)^11^. Non-metastatic patients across EC, GC, and CRC revealed equal proportions of CTC-positive patients (12.5%; 16.67%; 13.34%). In Fig. 2b-e the bar graph shows the percentage of CTC-negative (grey) vs. CTC-positive (orange) patients per tumor type. Following we further characterized the CTC-positive patients by their expression of PD-L1 in PD-L1^-^ and PD-L1^+^ CTCs (orange vs. red dots). However, in patients with known liver metastasis, the proportion of CTC-positive patients was significantly higher (28.57%) (Fig. 2b-e). Despite these parallels, the PD-L1 analysis revealed an uneven distribution of PD-L1^+^ CTCs. While in EC, the minority of CTCs expressed PD-L1, in both CTC-positive GC patients, PD-L1 was expressed on at least one cell (range: one out of two and eight out of 17) (Fig. 2b,c). In contrast, the non-metastatic CRC patients did not show any PD-L1 expression on their CTCs. Interestingly the majority of the M1 CRC patients with distant liver metastases showed PD-L1 expression in every second patient, respectively 50% with at least one PD-L1-positive CTC (range: one to five out of five) (Fig. 2d,e).

Accordingly, immunohistochemical analysis of PD-L1 expression in the tumor of 12 patients with PD-L1^+^ CTCs was performed. Despite the overall minor proportion of PD-L1^+^ tumor cells, the comparison revealed a slightly higher Tumor Proportion Score (TPS) of PD-L1^+^ tumor cells in the metastatic sides than the primary tumor sides (TPS CRC < 0.1% vs. TPS Mx 1–4%) (Fig. 2f,g). As a limiting factor these primary tumors and metastases do not represent matched samples. Overall, Tumor Proportion Scores (TPS) remained low in primary tumors, whereas PD-L1 expression on circulating tumor cells in patients with advanced disease approached 50%. These findings indicate that tissue-based PD-L1 assessment may have limited value in gastrointestinal cancers and support the pursuit of liquid biopsy–derived biomarkers such as CTCs. At the same time, the discrepancy underscores the chance to use PD-L1 expression on CTCs as a rationale for therapy rather than relying solely on PD-L1 expression in tumor tissue, as the two may reflect different aspects of tumor biology.

### PD-L1^+^ CTC patients show reduced immunocompetence of circulating anti-tumorigenic T-cells

In the blood, CTCs become exposed to immune surveillance and require manifold mechanisms to hide from the endogenous defense system. To this end, we first investigated the T-cell composition in the bloodstream of our CTC-positive and CTC-negative patients and especially analyzed the PD-1 status of the immune cells. Hence, for maximal resolution and realistic expression of the co-inhibitory receptor PD-1, T cells were characterized by surface-based staining and analyzed by multifactorial flow cytometry.

**Fig. 3:**
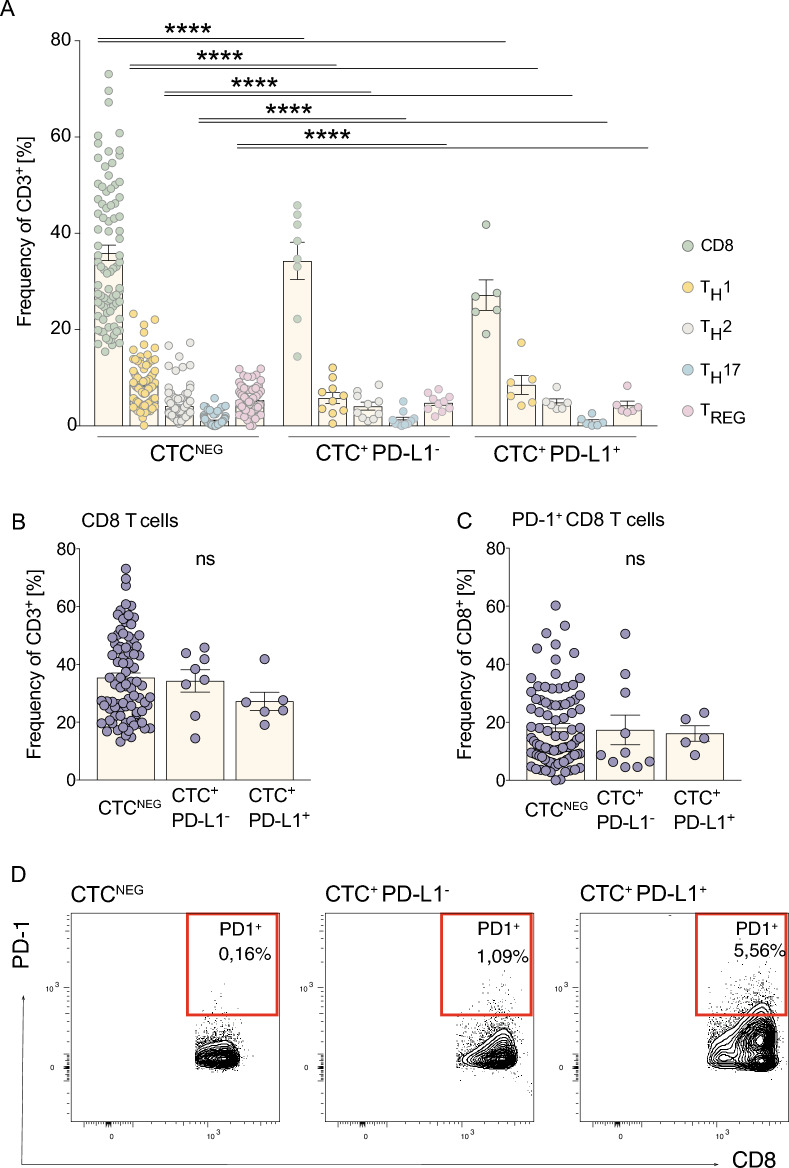
Circulating tumor cells (CTCs) and PD-L1 expression are associated with alterations in T-cell subset composition and CD8⁺ T-cell activation status. (**A**) Relative frequencies of CD3⁺ T-cell subsets, including TH1, TH17, TH2, regulatory T cells (TREG), and CD8⁺ T cells, in peripheral blood of CTC-negative patients compared with CTC-positive patients stratified by PD-L1 expression on CTCs (PD-L1⁻ versus PD-L1⁺). (**B**) Proportion of CD8⁺ T cells within the CD3⁺ T-cell compartment across the indicated patient groups, demonstrating differences associated with the presence of CTCs and their PD-L1 status. (**C**) Frequency of PD-1–expressing CD8⁺ T cells among CD3⁺ lymphocytes in CTC-negative patients and CTC-positive patients with PD-L1⁻ or PD-L1⁺ CTCs, reflecting differences in T-cell activation and potential exhaustion phenotypes. (**D**) Representative flow cytometry plots illustrating PD-1 expression on CD8⁺ T cells are shown below. Data are presented as frequencies (%). Each dot represents one individual patient. Horizontal lines indicate median values. Statistical significance was assessed using appropriate non-parametric tests (as described in Methods). ****P < 0.0001; ns, not significant.

Remarkably, we found significant differences in the overall frequencies of cytotoxic CD8^+^ T cells and effector CD4^+^ T cells within our cohort of cancer patients with or without detectable CTCs in their blood. Moreover, T cell composition was even significantly altered between patients with PD-L1^-^ CTCs or PD-L1^+^ CTCs, resulting in an overall reduction of the T cell compartment (Fig. 3a (ANOVA + Sidak’s mixed effect multiple comparisons: CD8^+^ T cells: CTC_NEG_ vs. CTC_POS PD-L1-_ or CTC_POS PD-L1+_ *p* < 0.0001; T_H_1: CTC_NEG_ vs. CTC_POS PD-L1-_ or CTC_POS PD-L1+_ p < 0.0001; T_H_2: CTC_NEG_ vs. CTC_POS PD-L1-_ or CTC_POS PD-L1+_ p < 0.0001; T_H_17: CTC_NEG_ vs. CTC_POS PD-L1-_ or CTC_POS PD-L1+_ *p* < 0.0001; T_REG_: CTC_NEG_ vs. CTC_POS PD-L1-_ or CTC_POS PD-L1+_ *p* < 0.0001)) and Supp Fig. 2 a,b).

In cancer defense, CD8^+^ T cells play a major role in detecting and degrading tumor cells. However, tumor cells can escape from the immune system by restraining the cytotoxic activity by ligation to PD-1. To estimate the proportion of anergic immune cells, we performed fluorescence-activated cell sorting especially for PD-1^+^ CD8^+^ T cells in the patient’s blood. While total CD8^+^ T cells were reduced in CTC harboring cancer patients, we detected a higher frequency of PD-1^+^ CD8^+^ T cells throughout CTC_POS_ patients, especially in patients with active PD-L1 expression onto the CTCs than among CTC_NEG_ patients (Fig. 3b–d). While our study observed significant differences in circulating T-cell subsets between patient groups, we did not perform functional assays such as cytokine profiling or cytotoxicity assessments. Therefore, our data reflects phenotypic observations of immune exhaustion markers but does not confirm functional impairment.

## Discussion

New immunotherapies, which aim to enable the endogenous immune system to fight cancer cells, have made great strides in cancer therapy in the past^12^. However, in the field of gastrointestinal tumors, therapeutic results were below expectations, and immune checkpoint blockade therapies were implemented in the oncological guidelines only as second-line therapy in advanced, already metastatic, patients^7^.

The most established biomarker for predictive therapeutic response is the expression of PD-L1 in the tumor tissue. However, reliable PD-L1 assessment on tumor tissue represents a major challenge. Its expression can be the result of a cancer cell-intrinsic genic process on the one hand but also is described as a reactive process to T cell response ^13–15^. Additionally, tissue staining of PD-L1 is technically challenging, leading to inhomogeneous results of PD-L1 positivity in tissues^16,17^. Moreover, pre-surgery biopsies display only a marginal percentage of the tumor site. Given the heterogeneity of PD-L1 expression and its staining, biopsies may lead to wrong assumptions and therapeutic consequences. All in all, PD-L1 status in tumor tissue appears to be an imperfect biomarker for clinical application. This underlines that early decision-making in the therapeutic approaches of gastrointestinal tumors is not available for immune therapies yet and predicting which patients will benefit from immunotherapy is not possible due to the lack of prognostic markers. These prognostic markers are desperately needed to identify patients, enable immunotherapy early, and improve patient outcomes.

The idea of liquid biopsies has been adequately investigated and suggests accurate prognostics through the detection of CTCs in many different entities, including CRC, EC, and GC ^18–20^. We and others have previously shown that CTC counts in the peripheral blood are associated with significantly worse outcomes^21,22^. In the present study, we aimed to evaluate the role of PD-L1 expression of CTCs isolated from the peripheral blood of gastrointestinal cancer patients. Our goal was to investigate whether the expression of PD-L1 provides additional prognostic information and justifies an early use of immunotherapeutic agents in specific patient groups. So far, PD-L1 expression on CTCs has been examined in tumor entities with first-line immunotherapies, for example, such as NSCLC or head and neck squamous cell carcinoma (HNSCC)^23,24^. A detailed examination of CTCs in gastrointestinal cancer patients is still missing.

In our study, we examined 105 patients with either esophageal, gastric, or colorectal cancer. 35.29% of the patients with CTCs in the bloodstream were expressing PD-L1, with levels ranging from 1 to 8 PDL1^+^ CTCs (Fig. 1d).

Here, we show that especially PD-L1^+^ CTCs are connected with advanced tumor stage and a higher likelihood of distant metastases and venous cancer spread in GI cancers, showing statistically significant correlation between PD-L1 expression and advanced TNM classification (PD-L1^+^ CTCs: pT_PD-L1+_ = 0.09; pM_PD-L1+_ = 0.01; pV_PD-L1+_ = 0.006) (number of PD-L1^+^ CTCs: pT_#PD-L1+_ = 0.02; pM_#PD-L1+_ = 0.007; pV_#PD-L1+_ = 0.0001) (Fig. 1 f). Pertinently, previously Satelli et al. showed that PD-L1^+^ CTCs were associated with shorter progression-free survival and reduced overall survival in prostate cancer and CRC^25^. Congruently, a similar trend was shown in patients with urothelial cancer^11^. Remarkably, our data indicates an increase of PD-L1^+^ CTCs in CRC patients with liver metastases compared to non-metastatic CRC patients (0% PD-L1^+^ CTC_CRC_ vs. 50% PD-L1^+^ CTC_MTx_) (Fig. 2d, e), no matter if the patients were treated with conventional (radio)chemotherapy or not. These findings let us assume that especially PD-L1^+^ CTCs have a better chance of survival in the blood and thus lead more frequently to distant metastases and disease progression. Taken together, these results underline the fact that CTCs are positively connected to the advanced tumor stage but also lead to the hypothesis that especially PD-L1^+^ CTCs facilitate the growth of distant metastases.

However, we could not detect a significantly higher expression of PD-L1 in the metastatic tissue compared to that of the primary tumor sites* (*Fig. 2f). In contrast to Ilié et al., who detected congruent expression of PD-L1 on CTCs and tumor tissue in advanced NSCL-cancer patients^24^. Given the fact that PD-L1 expression is overall low in CRC patients and staining results are heterogeneous, our results are limited and could change with the help of higher patient inclusion^26^. Instead, this emphasizes that in GI cancers the PD-L1 expression on CTCs is biologically more important as a prognostic marker as the overall low expression in the tumor tissues. One reason might be the overall immunosuppressed microenvironment in the tumor tissue with only little advantage through additional CIR expression. In contrast, in the blood, PD-1 expression helps to evade the humoral immune system. PD-1 expression on T cells can indicate either recent activation or functional exhaustion, depending on the cellular context and the co-expression of additional inhibitory checkpoint receptors such as LAG-3, TIM-3, and TIGIT^27^. At the present stage, however, a comprehensive analysis of the corresponding ligands other than PD-L1 was technically not feasible. For this reason, we focused our investigation on the PD-1/PD-L1 axis.

One possible mechanism and underlining of the biological relevance of PD-L1^+^ CTCs is their strong interaction with the blood’s immune cells, especially T cells. Here, we describe for the first time a strong correlation between the frequency of the circulatory T cells and the presence of CTCs (Fig. 3a). Interestingly, CTC^-^ cancer patients tend to have more T cells in the bloodstream for an ideal immune defense in comparison to cancer patients with PD-L1^-^, or respectively, PD-L1^+^ CTCs (Fig. 3a). Remarkably, this trend holds true for the cytotoxic CD8^+^ T cells, as well as CD4^+^ T cell compartments. Considering the PD-1/PD-L1 receptor-ligation axis and the subsequent T cell inactivation, we evaluated the PD-1 status of the cytotoxic cells. Nonetheless we did not perform functional assays to fully quantify the exhaustion of PD-1^+^ T-cells. Overall, our CTC^+^ patient collective displayed a higher proportion of inactive PD-1^+^ CD8^+^ T cells. However, their frequencies did not correlate significantly with PD-L1 expression onto the CTCs themselves, even though we could examine a trend of higher PD-1 expression on CD8 T cells in CTC^+^ PD-L1^+^ patients (Fig. 3b-d). Congruently, Ilié et al. showed a concordance of PD-1 expression on overall white blood cell count with PD-L1^+^ CTCs^24^. However, the white blood cell count is very unspecific for an anti-tumor response. In the defense of cancer cells, normally, the cytotoxic CD8^+^ T cells play a major role. A receptor ligation of PD-1 by the PD-L1^+^ cancer cells leads to an anergic status of these cells, resulting in the disability to kill the cancer cells. Therefore, we untangle the different T-cell compartments of the blood, concluding that cancer patients with PD-L1^+^ CTCs have an attenuated immune defense due to firstly a reduced frequency of T cells and secondly a relatively higher proportion of inactive CD8^+^ T cells in the blood.

However, this study presents several limitations, as the small number of CTC-positive patients and PD-L1^+^ CTCs constraining statistical power and limits the extrapolation of our findings. Moreover, an important limitation of this study is the heterogeneity of the patient cohort, which included different tumor types as well as both treatment-naïve patients and patients who had received neoadjuvant chemotherapy or radiochemotherapy. Although no significant association between neoadjuvant treatment and CTC or PD-L1 + CTC detection was observed, treatment-related effects on tumor biology and immune status cannot be fully excluded.

Additionally, functional validation of immune cell activity was not performed, restricting our conclusions on immune exhaustion to phenotypic observations. Finally, the absence of survival or longitudinal outcome data prevents us from evaluating the true prognostic significance of PD-L1^+^ CTC detection.

In conclusion, our findings suggest a potential association between PD-L1-positive circulating tumor cells, advanced disease stage, and altered systemic immune profiles in gastrointestinal cancer patients. However, these exploratory results require validation in larger, multicenter cohorts using standardized PD-L1 detection methods and robust statistical analyses, including survival outcomes. Until then, PD-L1^+^ CTCs should be viewed as an intriguing but still unvalidated prognostic biomarker, with potential relevance for immunotherapy stratification.

## Materials and Methods

### Study design

The present study was conducted as a single-center study with 105 enrolled patients, diagnosed with histologically proven esophageal (48), gastric (6), and colorectal cancer (30), as well as 21 liver metastases of colorectal cancer (Table 2). Patients were enrolled consecutively between 06/2021 and 06/2022.

The study was approved by the Medical Ethical Committee, Hamburg, Germany, and complies with the principles of the Declaration of Helsinki (Ethical approval number: PV5348). Informed consent was obtained from all patients. All enrolled patients of this study were initially considered resectable and underwent surgery at the Department of General, Visceral and Thoracic Surgery at the University Medical Center Hamburg-Eppendorf.

69 of the patients received neoadjuvant (radio-)chemotherapeutic treatment and one of the patients was treated with anti-PD1 targeted immunotherapy. Peripheral blood samples for CTC and PBMC were collected immediately before surgery. Demographic, clinical, operative, and postoperative data were collected for every patient.

Histopathological analyses were performed by specialists for gastrointestinal pathology at the Institute of Pathology of the University Medical Center Hamburg-Eppendorf. All resected lymph nodes (LN) were counted, identified by location, and assessed separately. Standard histopathological analysis of paraffin-embedded LNs was performed by preparing 5–μm–thick serial sections, hematoxylin–eosin staining, and van Gieson staining. Tumor type, stage, and grade were analyzed and evaluated according to the seventh edition of the tumor, node, and metastasis classification^28^. Studies of tumor markers (carcinoembryonic antigen and cancer antigen 19–9) and bone scans were also performed for all patients.

### CTC analysis

CTC analysis was performed using the CellSearch system and the CXC kit as previously described^11,29,30^. Blood samples (7.5 mL) were collected in CellSave preservative tubes, stored at room temperature, and processed within 48 h, according to the manufacturer’s instructions. The presence of a nucleus, cytokeratin expression, round or oval cell morphology, and absence of CD45 expression were the criteria for CTCs^29^. The cut-off value for CTC positivity was 1 CTC/7,5 mL)^30,31^.

### Determination of PD-L1 expression of CTCs

PD-L1 expression of CTCs was analyzed in the CellSearch system using the CellSearch CXC Kit and the (Menarini, Silicon Biosystems, Bologna, Italy) free fourth channel for phycoerythrin (PE). The anti-PD-L1-antibodies (E1L3N XP rabbit mAb, PE conjugate, or D8T4X, PE conjugate, Cell Signaling Technology [CST]) were diluted in Antibody Diluent (DAKO Cytomation) within Menarini-supplied reagent cups (concentration 1:50, final concentration in device 1:237). PD-L1-specific immunofluorescence of CTCs was evaluated after an exposure time of 1.6 s and categorized into negative (0), weakly positive (1 +), moderately (2 +) and strongly positive (3 +), using cross validated antibody intensity^11^. Since the intensity of PD-L1 immunofluorescence of CTCs was generally low, PD-L1^+^ CTCs were defined as CTCs with any intensity of PD-L1 staining.

### PBMC analysis

The blood samples were processed for lymphocyte isolation immediately. Whole blood samples were diluted with equal amounts of PBS, followed by Pancoll (PAN-Biotech) gradient separation. For extracellular staining, cells were re-suspended in 100 μl MACS buffer (1 × PBS, 1% FBS, 0.5% EDTA) containing directly fluorochrome-labeled antibodies and incubated for 20 min at 4 °C in the dark. Cells were washed and re-suspended in 300 μl of MACS buffer for direct acquisition with BD FACSymphony A3.

FACS panel: Fixable Viability Dye eFluor (BV510, ThermoFisher, Dilution 1:1000), CD45RO/RA (AlexaFluor700, BioLegend, Dilution 1:100), CD4 (BUV395, BD Biosciences, Dilution 1:100), CD8 (APCCy7, BioLegend, Dilution 1:400), IL-17A (BV711, BioLegend, Dilution 1:200), CD25 (BV650, BioLegend, Dilution 1:100), CD127 (FITC, BioLegend, Dilution 1:100), CCR4 (BV605, BioLegend, Dilution 1:200), CCR6 (APC, BioLegend, Dilution 1:200), CXCR3 (PEDazzle, BioLegend, Dilution 1:200), CD3 (BUV737, BD Biosciences, Dilution 1:100), PD-1 (BV421, BioLegend, Dilution 1:400), CTLA-4 (PeCy7, BioLegend, Dilution 1:100).

### Histopathological staining

PD-L1 expression in tumor cells as well as in the accompanying inflammatory infiltrate was assessed by immunohistochemistry using the antibody MSVA-711R (syn. MSVA-011, MS Validated Antibodies). MSVA-711R showed the same performance characteristics as anti-PD-L1 CST E1L3N in comparative studies^35,36^ and was evaluated according to identical criteria: The Tumor Proportion Score (TPS) describes the ratio of vital PD-L1 positive tumor cells to all vital tumor cells. Immune Cell-Scoring (IC-Scoring) determines the percentage of tumor area occupied by PD-L1 positive immune cells. The Combined Positivity Score (CPS) takes into account both PD-L1 positive tumor cells and PD-L1 positive mononuclear inflammatory cells and is expressed in terms of all tumor cells.

### Statistical analysis

PASW Statistics 18 software (SPSS Inc., Chicago, IL) was used. Histological characteristics were expressed as descriptive statistics. The χ^2^ test was used to investigate the association between CTCs, PBMCs, and histopathological parameters. The correlation plots were generated using the R statistical computing environment. (R version 4.2.1. https://cran.r-project.org/web/packages/corrplot/vignettes/corrplot-intro.html and the CRAN package corrplot (version 0.95) (Taiyun Wei and Viliam Simko (2024). R package ‘corrplot’: Visualization of a Correlation Matrix (Version 0.95). Available from https://github.com/taiyun/corrplot). The correlation matrix (M) was computed using Pearson correlation coefficients based on the processed descriptor dataset. Statistical significance (p-values) for correlations was calculated using the cor.mtest() function to generate the corresponding p-value matrix.

## Supplementary Information

Supplementary Information 1.

Supplementary Information 2.

## Data Availability

He datasets used and/or analyzed during the current study are available from the corresponding author on reasonable request.

## Abbreviations

CRC: Colorectal cancer
CTC: Circulating tumor cell
CIR: Coinhibitory receptors
EC: Esophageal cancer
GC: Gastric cancer
GI: Gastrointestinal
HNSCC: Head and neck squamous cell carcinoma
MTx: Metastases
NSCLC: Non-small cell lung cancer
PBMC: Peripheral blood mononuclear cell
PD-1: Programmed cell death protein 1
PD-L1: Programmed cell death protein–ligand 1
TPS: Tissue polypeptide-specific antigen
