# Supplementary material for: PD-L1-positive circulating tumor cells associate with tumor malignancy and impaired circulating immunity in patients with gastrointestinal tumors
Source: Sci Rep. 2026 May 7;16:15870. doi: 10.1038/s41598-026-43324-y (PMC13194882; doi:10.1038/s41598-026-43324-y)
Supplement: Supplementary file 1 — Supplementary Information 1. [file 41598_2026_43324_MOESM1_ESM.docx]

**Supplementary Fig 1:** **(A)** Statistical correlation (χ^2^ test) of circulating tumor cell (CTC) positivity with key clinical parameters, including tumor stage, patient age, gender, neoadjuvant chemotherapy, neoadjuvant radiotherapy, and tumor markers (CA 19-9, CEA). The heatmap visualizes the relationships between these factors across different patient groups. **(B)** Statistical correlation (χ^2^ test) of CTC positivity and PD-L1 expression in esophageal cancer patients with clinical and molecular parameters, including tumor stage, gender, age, HER2 mutation, MSI status, immune infiltration, CA 19-9 (pre- and post-operation), CEA (pre-operation), and neoadjuvant chemotherapy or radio chemotherapy. **(C)** Statistical correlation (χ^2^ test) of CTC positivity and PD-L1 expression in gastric cancer patients with clinical and molecular parameters, including tumor stage, gender, age, HER2 mutation, MSI status, immune infiltration, CA 19-9 (pre- and post-operation), CEA (pre-operation), and neoadjuvant chemotherapy. **(D)** Statistical correlation (χ^2^ test) of CTC positivity and PD-L1 expression in colorectal cancer patients (with or without liver metastasis) with clinical and molecular parameters, including tumor stage, gender, age, HER2 mutation, MSI status, KRAS and BRAF mutations, immune infiltration, CA 19-9 (pre- and post-operation), CEA (pre- and post-operation), neoadjuvant chemotherapy and neoadjuvant radio chemotherapy.

**Supplementary Fig 2: (A)** Flow cytometry gating strategy for the identification of lymphocyte subpopulations, including CD3^+^ T cells, CD4^+^ and CD8^+^ T cells, PD-1^+^ CD8^+^ T cells, and CD4^+^ T-cell subsets (T_REG_, T_H_1, T_H_2, T_H_17). **(B)** Statistical correlation (χ^2^ test) comparing tumor entity, circulating tumor cell (CTC) positivity, CTC count, and PD-L1^+^ CTCs with immune cell populations. These include CD8^+^ T cells and CD4^+^ T cells with regulatory T cells (T_REG_), T_H_1, T_H_2, T_H_17 subsets. The heatmap illustrates the relationships between CTC characteristics and immune cell composition in peripheral blood mononuclear cells (PBMCs).
