# Supplementary figures and images for: PD-L1-positive circulating tumor cells associate with tumor malignancy and impaired circulating immunity in patients with gastrointestinal tumors
Source: Sci Rep. 2026 May 7;16:15870. doi: 10.1038/s41598-026-43324-y (PMC13194882; doi:10.1038/s41598-026-43324-y)

A

All

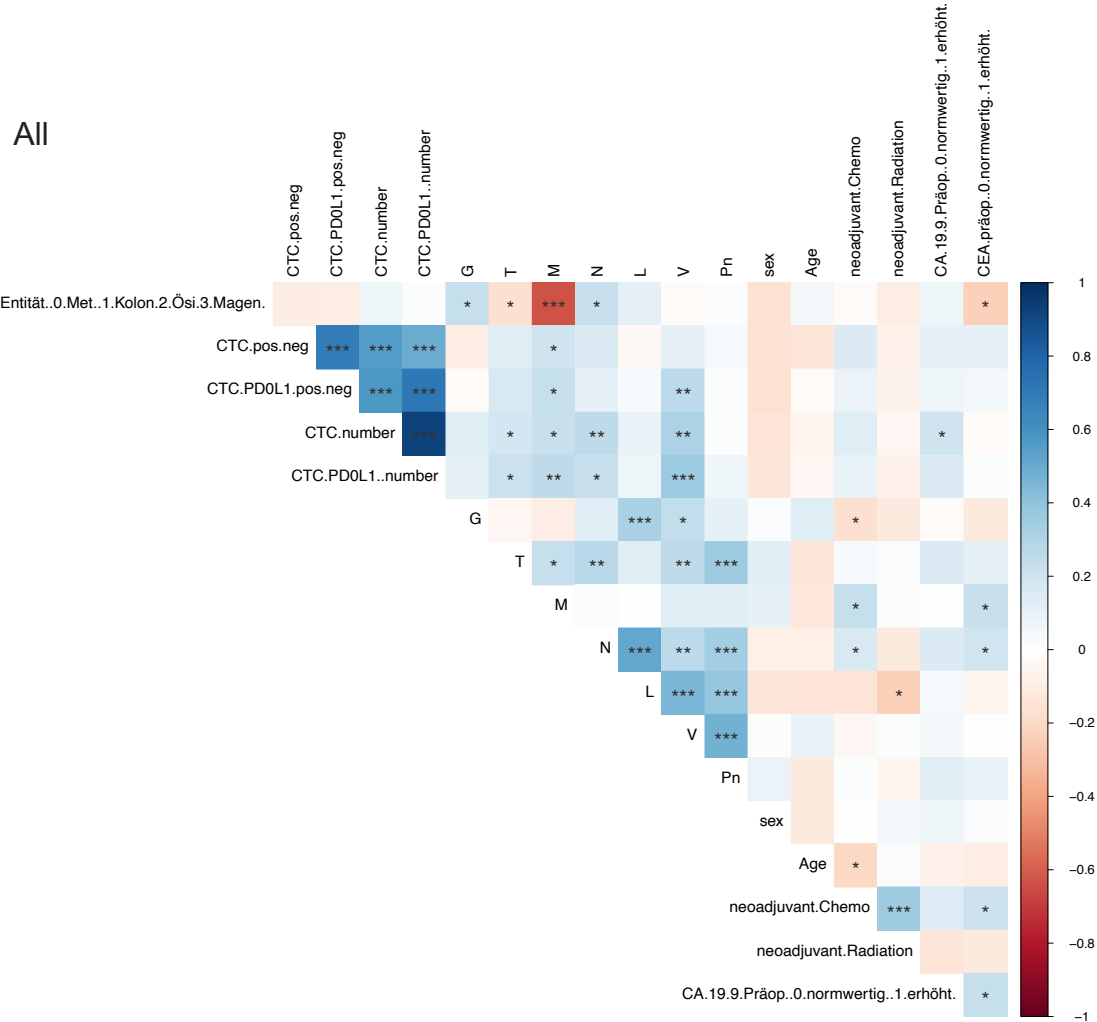

B

Esophageal Cancer

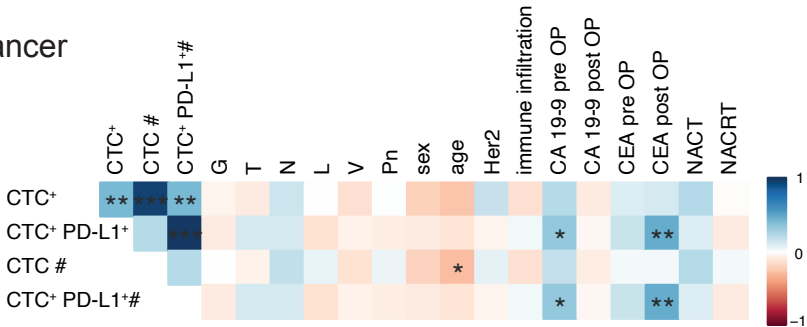

C

Gastric Cancer

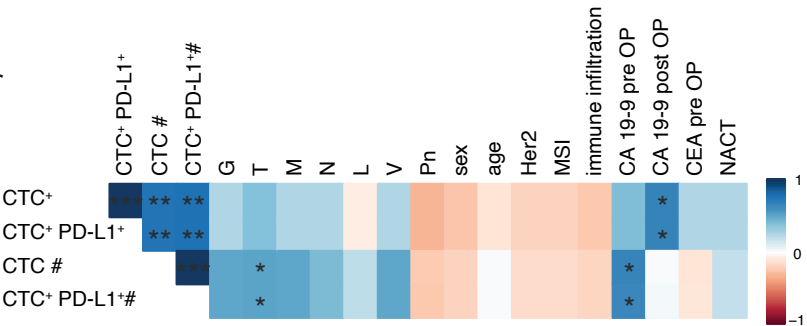

D

CRC + MTx

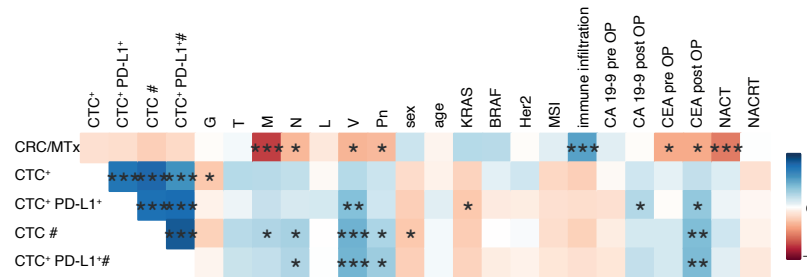

Supplementary Figure 1

A

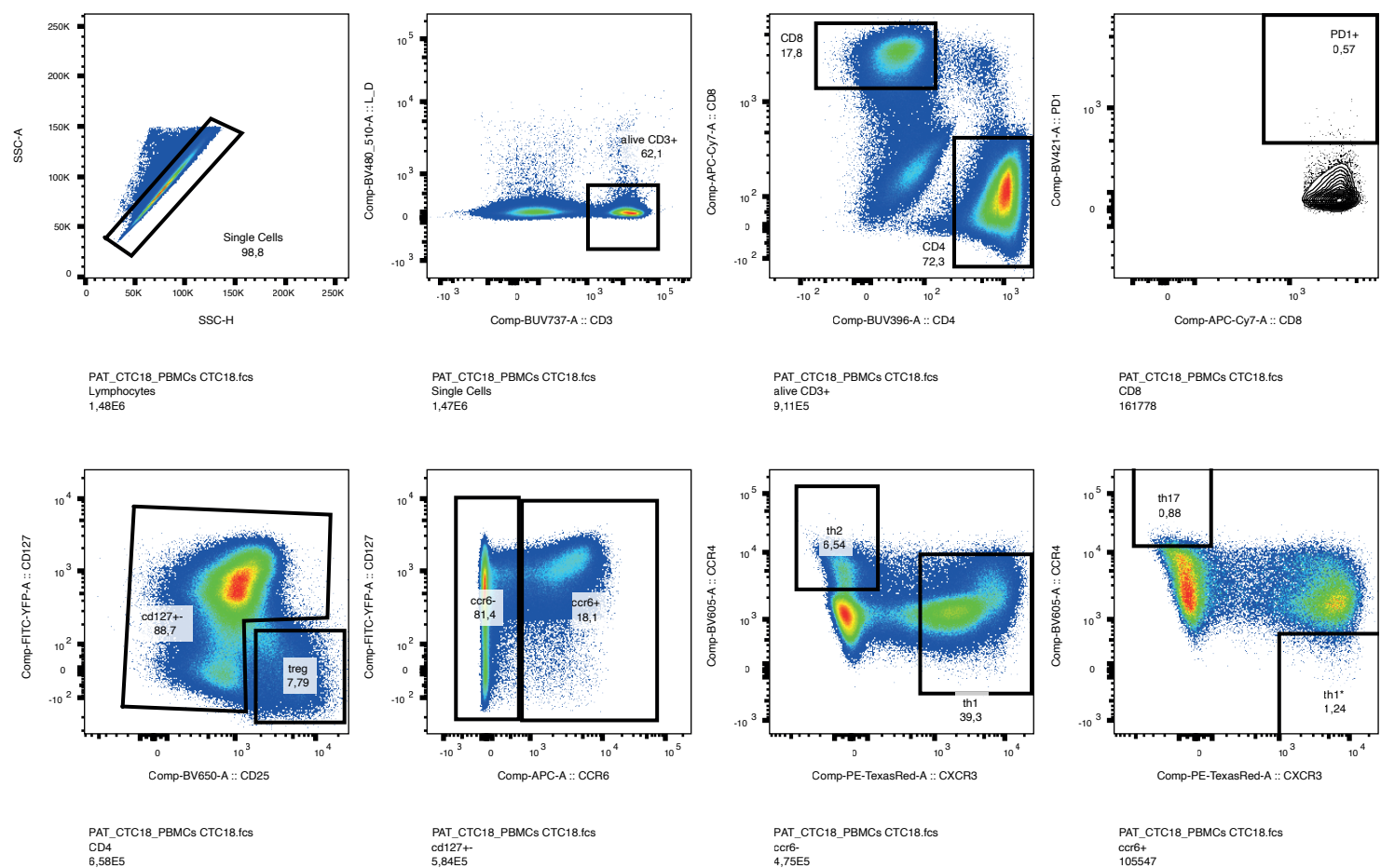

B

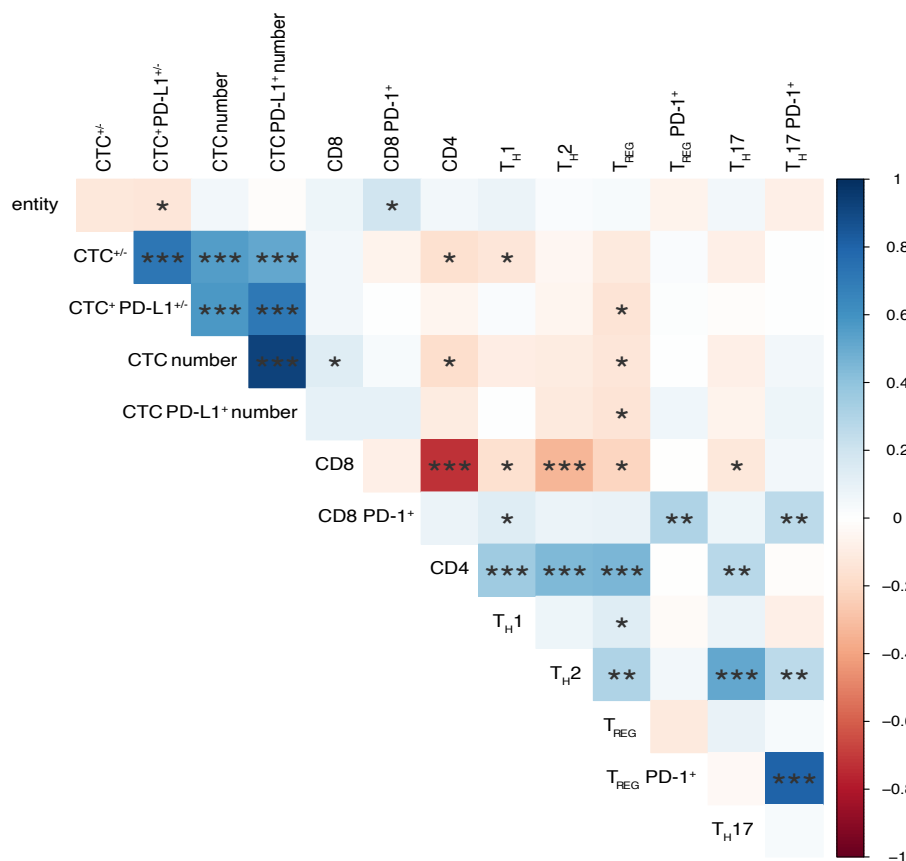

Supplement: Supplementary file 2 — Supplementary Information 2. [file 41598_2026_43324_MOESM2_ESM.pdf]
